# 

**Published:** 1889-05-18

**Authors:** 


SATURDAY, MAY 18th, 1889.

**Neutral Science** - - - 95
**The New Electric Tramcars** - - - 96
**Hospitals and their Expenditure** - - - 97
**Should Out-Patients Pay?** - - - 98
**The Donegal Fund** - - - 98
**The Doctor's Pulpit.**—Man and his Enemies - - - 99
**M. Zola on French Journalism and its Health Results.** By Col. Bowness Fischer - - - 100
**Within the Wards.**—Another Cancer Cure—Scrofulus Glands—A Lung Cavity opened Surgically - - - 101
**Notes and News** - - - 103
**Everybody's Page** - - - 104
**Annotations.**—"Cuckoo!"—A Magisterial Valuation of Child-Life—Carlsbad - - - 105
**Turning Over New Leaves.**—Board School Laryngitis - 106
**National Eye and Ear Infirmary, Dublin** - - - 106
**"Her Heart's Mistake."** By E. D. Cumming, Author of "An Ordeal by Silence" - - - 107
**The Working Classes and the Hospitals.** By Mr. B. Burford Rawlings - - - 109
**Father Damien.—A Life given to the Lepers** - - 109
**Amusements—Scraps and Gleanings** - - - 110

**EXTRA SUPPLEMENT—THE NURSING MIRROR.**

**En Passant.**—Fitzroy House—Sad News from India—Youthful Nurses—Short Items—Massage—Westminster News—At the Academy - - - xxiii
**Notes from Australia** - - - xxiv
**Nursing in the Soudan.**—The Victoria Hospital - xxiv
**Lectures** - - - xxv
**Everybody's Opinion.**—The British Nurses Association—A Hint to Midwives - - - xxv
**Vacancies** - - - xxvi
**Notes and Queries** - - - xxvi

